# Current State of Analgesia and Sedation in the Pediatric Intensive Care Unit

**DOI:** 10.3390/jcm10091847

**Published:** 2021-04-23

**Authors:** Chinyere Egbuta, Keira P. Mason

**Affiliations:** Department of Anesthesiology, Critical Care and Pain Medicine, Harvard Medical School, Boston Children’s Hospital, 300 Longwood Ave., Boston, MA 02115, USA; Chinyere.Egbuta@childrens.harvard.edu

**Keywords:** analgesia, sedation, PICU, critically ill pediatric patient, PICU-acquired complications, delirium, withdrawal

## Abstract

Critically ill pediatric patients often require complex medical procedures as well as invasive testing and monitoring which tend to be painful and anxiety-provoking, necessitating the provision of analgesia and sedation to reduce stress response. Achieving the optimal combination of adequate analgesia and appropriate sedation can be quite challenging in a patient population with a wide spectrum of ages, sizes, and developmental stages. The added complexities of critical illness in the pediatric population such as evolving pathophysiology, impaired organ function, as well as altered pharmacodynamics and pharmacokinetics must be considered. Undersedation leaves patients at risk of physical and psychological stress which may have significant long term consequences. Oversedation, on the other hand, leaves the patient at risk of needing prolonged respiratory, specifically mechanical ventilator, support, prolonged ICU stay and hospital admission, and higher risk of untoward effects of analgosedative agents. Both undersedation and oversedation put critically ill pediatric patients at high risk of developing PICU-acquired complications (PACs) like delirium, withdrawal syndrome, neuromuscular atrophy and weakness, post-traumatic stress disorder, and poor rehabilitation. Optimal analgesia and sedation is dependent on continuous patient assessment with appropriately validated tools that help guide the titration of analgosedative agents to effect. Bundled interventions that emphasize minimizing benzodiazepines, screening for delirium frequently, avoiding physical and chemical restraints thereby allowing for greater mobility, and promoting adequate and proper sleep will disrupt the PICU culture of immobility and reduce the incidence of PACs.

## 1. Introduction

Despite an increase in acuity and medical complexity of pediatric patients in the intensive care unit, the last two decades have demonstrated a significant drop in patient mortality [1,2]. This success has been in large part a consequence of technological advancements in physiological monitoring and interpretation, invasive testing, and complex medical procedures, many of which usually require a combination of analgesia, anxiolysis, amnesia, and sedation [3,4]. Balancing this combination of analgesia and sedation is challenging for this diverse spectrum of age, maturity (developmental and emotional), and medical complexity [5]. Currently, the incidence of PICU acquired complications (PAC) outnumbers the rate of PICU mortality. Improvements in sedation delivery have been shown to decrease the incidence of physical restraints, post-traumatic stress disorder, oversedation, delirium, and neuromuscular weakness [6].

This review will present all the most up-to-date and relevant literature that addresses important aspects of PICU (and adult-related when relevant) sedation: national and international sedation guidelines, pharmacodynamics and pharmacokinetics of analgosedative drugs, sedation and analgesia assessment rubrics, pain management, goal-directed sedation strategies, adverse events and side effects, the need for neuromuscular blockade, and optimal analgesic and sedation practices. The authors searched PubMed, Medline, and the Boston Children’s Hospital medical e-library using the following search terms: sedation in critically ill patients/pain management in the intensive care unit (ICU)/current practices in analgesia and sedation solely at first, then further specifying pediatric patients. The authors also used supplementary search methods such as assessing the similar articles section in PubMed search results and the reference lists of selected studies. The authors included both pediatric and adult studies that are most relevant to clinical practice, while highlighting studies that were published after 2000. Understanding the factors involved in attaining the right balance of adequate analgesia and optimal sedation guided by therapeutic targets that evolve with the changes in each patient’s medical condition is key to reducing PACs as well as improving clinical outcomes and functional recovery in critically ill pediatric patients [6].

### 1.1. National and International Analgesia and Sedation Guidelines

In 2006, the United Kingdom Paediatric Intensive Care Society Sedation, Analgesia and Neuromuscular Blockade Working Group, a multi-disciplinary expert panel, used a modified Delphi technique to create consensus guidelines on sedation, analgesia, and neuromuscular blockade in critically ill children. The panel warned that the quality of evidence available in the literature to support these recommendations is poor and that there is little evidence to guide PICU staff with the common clinical problems of tolerance, withdrawal, and the patient who requires long-term sedation or who are difficult to sedate with standard agents [7,8]. In 2016, a multidisciplinary group of experts from members of the European Society of Paediatric and Neonatal Intensive Care (ESPNIC) produced a position statement to help guide professionals in the assessment and reassessment of the effectiveness of treatment interventions for pain, distress, inadequate sedation, withdrawal syndrome, and delirium [9].

A retrospective study evaluated the pharmaceutical management of sedation, analgesia, and neuromuscular blockade medications administered to 66,343 children in 161 ICUs in the United States between 2009 and 2016. The durations of opiate and sedative administration were associated with predicted complications of tolerance and withdrawal. Several medications were dispensed in conflict with Food and Drug Administration (FDA) warnings [10]. Surveys of analgosedative practices in PICUs have demonstrated a wide variability in clinical practice typically associated with individual provider preference, local culture, the use of multiple drug combinations and classes, variations in dosing and routes of administration, as well as the use of off-label drugs [11,12,13,14]. The relative scarcity of high quality pediatric studies and randomized controlled trials in the area of analgosedation makes it challenging to develop consensus guidelines and best practice recommendations.

### 1.2. Pharmacokinetics and Pharmacodynamics in Critically Ill Children

Pharmacokinetics (PK) and Pharmacodynamics (PD) are important when considering sedation and analgesia, particularly as the clearance, metabolism, and duration of effect can be impacted by end-organ (hepatic, renal, cardiac) failure and dysfunction [15,16]. The key factors that impact PK and PD in the PICU patient can be divided into two elements: Patient Factors and PICU Factors. Patient factors include body fluid shifts that impact drug volume of distribution; altered protein binding that can impact plasma concentration of drugs; end organ dysfunction that can alter drug absorption, metabolism, and excretion; natural age-related physiology that can impact drug absorption, metabolism, and excretion; and inflammatory states that alter drug-metabolizing enzymes and transporters and impact drug absorption, efficacy, and clearance [17,18,19,20,21]. PICU factors include non-pharmacologic interventions such as continuous renal replacement therapy (CRRT), extra-corporeal membrane oxygenation (ECMO), and therapeutic hypothermia, all of which affect volume of distribution, metabolism, absorption, and clearance [17,18,19,20,21,22,23,24,25].

Recent advancements in neonatal resuscitation combined with a significant decline in mortality introduce complex combinations of baseline multi-organ dysfunction which complicate the pharmacokinetic and pharmacodynamic profile of sedatives and analgesics in the PICU setting. As a result of the increasing complexity of interactions between the sedatives and the individual response, PICU sedation has evolved to encompass a special area of expertise [15,21].

## 2. Analgesia

Effective and tailored acute, procedural, and chronic pain management is critical in the PICU. These conditions are often overlapping and require acute sensitivity to different components. The goal of analgesic therapy is to provide comfort, reduce physiological stress response, and minimize analgesic-associated adverse events such as respiratory depression, risk of addiction, hemodynamic instability, and end organ injury. In the severely critically ill pediatric patient, this balance is a delicate one that, if unsuccessful, can subject children to inadequate pain management [15,26].

### 2.1. Pain Assessment

In order to titrate analgesic therapy to effect, manage pain adequately, and monitor for signs of medication toxicity or adverse effects, pain assessment is of utmost importance. Self-assessment to report pain scores, although considered the gold standard for monitoring the efficacy of analgesic therapy, is largely not possible for the majority of PICU patients [15,27]. Physiologic indicators (tachycardia, rise in blood pressure, tachypnea, pupillary dilatation, increased muscle tone, sweating, etc.) are equally unreliable markers as they lack sensitivity and specificity to pain. Pain-related behaviors, distinguished by verbal (vocalized description of intensity, quality, location with concomitant displays of moaning or crying) and non-verbal (facial expression, body posturing/repositioning, decrease in activity) cues have been shown to be similarly unreliable [27,28].

In children <3 years of age, despite the limitations and challenges to assessing and interpreting the physiological indicators of pain and pain-related behaviors, behavioral observation scales are the standard of care. Subjective and objective indicators are used to interpret facial expression and physiologic and motor responses, frequently engaging the opinions of family members and primary caregivers [15,27]. Children ages 4 to 8 years are usually able to self-report pain, facilitating the practice of matching their subjective feedback to a developmentally appropriate pain tool [15,29,30,31]. Older children (8 years and above), are typically able to give a self-assessment of their pain using more validated methods such as a verbal rating scale, numeric rating scale (NRS), and the Visual analog Scale (VAS), similar to adults [27,30]. Not all pain scales require self-reporting and interpretation of verbal cues. The Wong-Baker FACES scale and the Bieri Faces Pain Scale Revised (FPS-R) are suitable for children of any age and developmental stage and rely on non-verbal cues [30]. Each scale has its limitations. The Neonatal Pain, Agitation, and Sedation Scale (N-PASS), Non-Verbal Pain Scale (NVPS), and the Face, Legs, Activity, Cry, and Consolability scale (FLACC) are observational scales that are unable to quantify the intensity or quality of pain. Regardless, especially for sedated and pharmacologically paralyzed PICU patients, these scales are a valuable means to identify the presence of pain [30,32].

### 2.2. Systemic Analgesia: Opiates and Non-Opiates

#### 2.2.1. Opioids

Opioids work on opiate receptors which are found in the brain, spinal cord, and peripheral tissue: mu (µ) receptors (analgesia, euphoria, miosis, respiratory depression), kappa (k) receptors (spinal analgesia), sigma (σ) (dysphoria, respiratory and vasomotor stimulation, and hallucinations), and delta (Δ) (unclear effects) [27,33,34]. All opioids exhibit a dose-dependent respiratory depression which increases in risk when given in conjunction with other sedatives, commonly benzodiazepines [34,35]. Due to the risk of dependence and withdrawal, opiates should be weaned slowly in those patients who have received opioid infusions ≥7 days [33,34]. In general, in the PICU setting, opiates are favored for the relief of severe pain, especially perioperative and non-neuropathic. The Collaborative Pediatric Critical Care Research Network (CPCCRN) performed a prospective, observational study (the MOTIF (Measuring Opioid Tolerance Induced by Fentanyl (or morphine)) to characterize the exposure to opioid analgesia among mechanically ventilated children. Mechanically ventilated children required increasing opioid doses, often associated with prolonged opioid exposure or the need for additional sedation [36].

##### Morphine

Discovered over 200 years ago, morphine is the oldest of the opiates and the only hydrophilic opioid still in common use. A typical bolus dose of morphine achieves a peak effect in 10 to 20 min and has a duration of action of approximately 2–4 h [21,27]. Morphine is metabolized by the liver via glucuronidation and its metabolites are then renally excreted [37,38]. The active metabolite of morphine, morphine-6-glucuronide (10%), stimulates the mu-receptors, and it is recommended that especially in patients with renal insufficiency dosing of morphine should be carefully titrated or avoided altogether in order to avoid oversedation and respiratory depression [21,34,39,40,41]. Dosing of morphine must be done with consideration of the age, physiology, and medical condition of the child, as the pharmacokinetics of morphine differ from premature infants through childhood. Neonates <10 days old require less than half the dose of older children to attain similar plasma levels of morphine with similar analgesia [21,27,42]. Morphine, still the most commonly used sedative in the PICU, has important risks and side effects: vasodilation, hypotension, bronchospasm, and pruritus should be considered, however they are not usually clinically significant [21,27,30,34,42].

##### Fentanyl

Fentanyl is a synthetic morphine derivative that is highly lipophilic and fat soluble, over 100 times more potent than morphine and exhibits very quick onset (<1 to 2 min) and up to 60-min duration with intermittent doses [27]. With continuous prolonged administration, fentanyl can accumulate in peripheral compartments, increasing the context sensitive half-life and prolonging sedation [27,34]. Unlike morphine, fentanyl has an inactive metabolite, norfentanyl, that does not cause histamine release. Be aware that because fentanyl depresses the heart rate response, it can have a negative effect on children who are heart rate dependent and rely on increased heart rate for augmentation of cardiac output. Conversely, fentanyl has its advantages, particularly for blunting heart rate response to intubation and laryngoscopy [43,44,45,46,47]. Although rare, there is a risk of chest wall rigidity, usually with rapid large (>5 mcg/kg) doses, that can precipitate respiratory failure and necessitate intubation or naloxone [5,21,48].

##### Remifentanil

Remifentanil is a newer synthetic opioid that is equipotent to fentanyl and has an ultra-short half-life of about 3 to 4 min. Metabolized by plasma esterases, it does not accumulate and demonstrates a small volume of distribution [27]. Remifentanil is optimal for the patient with renal or hepatic dysfunction, as it avoids the risk of accumulation of active metabolites, and prolonged context sensitive half-life and duration of action. Remifentanil can facilitate the abrupt need to perform neurological exams, quickly wearing off even after prolonged infusions. This rapid onset and offset favors its use in the PICU setting, supporting rapid titration of analgesic and anesthetic depth with minimal fluctuations in hemodynamics [49]. Similar to other opioids, remifentanil does have respiratory and myocardial depressant effects that should be anticipated and managed appropriately. There has been concern that the ultra-short acting nature of remifentanil leads to an increased risk of rapid developmental of tolerance; also, it has been suggested that remifentanil has the highest association with opioid-induced hyperalgesia (OIH) amongst the opiates and OIH leads to prolonged post-operative recovery, increased length of stay, and significant discomfort [49,50]. The RAPIP trial (remifentanil-based analgesia and sedation of pediatric intensive care patients), a randomized controlled trial which aimed to compare remifentanil and fentanyl concerning the incidence of tolerance, withdrawal, and OIH, demonstrated no association between remifentanil and increased risk for tolerance, withdrawal or OIH [51].

##### Hydromorphone

Hydromorphone is a semi-synthetic opioid (a hydrogenated ketone of morphine) that selectively binds to the mu receptor [52]. Hydromorphone is hydrophilic and has 7 to 10 times the potency of morphine. Hydromorphone has an onset of 5 to 10 min and a duration of action of 3 to 4 h, similar in profile to morphine’s. Metabolized by hepatic glucuronidation to hydromorphone-3-glucuronide and excreted in the urine, hydromorphone is associated with less sedation, nausea, and pruritus than morphine [5,30,37,38]. Continuous infusions of hydromorphone have been shown to be effective for prolonged (greater than 24 h) sedation in the PICU setting. With a mean starting dose of 0.024 mg/kg/h, and a maximum mean dose of 0.05 mg/kg/h, 66% of mean daily FLACC scores were <1 in PICU patients sedated for a mean of 182 h [53].

##### Methadone

Methadone is a synthetic mu-receptor opioid agonist with rapid onset (5–10 min and 30–60 min via IV and oral route respectively). It has the longest duration of action of all the opioids (4 to 24 h). Methadone’s lack of known active metabolites, high oral bioavailability, and long duration of action supports its frequent choice to prevent opioid withdrawal for children who have received continuous morphine or fentanyl for >5 days [54,55,56]. Enteral administration of methadone has been shown to expedite opioid discontinuation and reduce the risk of withdrawal in critically ill children at very high risk for opioid abstinence syndrome [54,55,56,57].

The pharmacokinetic profile of methadone complicates an easy conversion to equipotent dose ratios between narcotics. There is still no consensus on an ideal conversion factor after prolonged IV fentanyl [54,58,59,60]. Methadone dose equivalents of 2.5 times the daily fentanyl dose have demonstrated success for weaning [48]. Applying standard adult dose conversion guidelines (23.7 mg oral morphine to 1 mg oral methadone) to the pediatric population, 53.7% of children in the PICU were successfully converted to methadone and 41.7% appeared undermedicated [61]. Regardless of the conversion formula applied, it is recommended that the Withdrawal Assessment Tool (WAT-1) and State Behavioral Scale be performed daily, to guide opioid weaning schedules [62,63].

Methadone carries a Food and Drug Administration (FDA) Boxed Warning due to its potential to prolong the corrected QT interval, potentially leading to life-threatening arrhythmias. A retrospective study of 51 PICU patients <18 years of age did not report an incidence of significant change of the corrected QT interval in this group. However, those that did manifest an increase in corrected QT interval of ≤40 ms, had structural heart disease. Caution should therefore be taken when administering methadone to PICU patients with structural heart disease [64]. It is important to note that methadone is gaining notoriety in the media as the premier agent for medication-assisted treatment of opioid addiction. Parents will frequently raise concerns over its use.

#### 2.2.2. Patient-Controlled Analgesia (PCA) and Parent/Nursing-Controlled Analgesia (PNCA)

PCA is a patient-controlled, on-demand, and intermittent means of administering sedatives and analgesics. PNCA is reserved for those patients who do not have the ability to control their self-administration. In these cases, the nurse or health care provider, often aided by the parent’s input, assume responsibility for the dosing. The relationship between meperidine blood concentrations and minimum analgesic concentration of meperidine for post-operative analgesia was discovered and used as the pharmacologic basis of PCA [65,66]. Minimum effective analgesic concentration (MEAC) is defined as the smallest concentration at which pain is relieved. PCA has evolved to include patient-controlled epidural analgesia (PCEA) or Epidural PCA, peripheral nerve catheter PCA or patient-controlled regional analgesia (PCRA), as well as a non-invasive method of transdermal PCA which delivers ionizable medications (fentanyl, for example) through the skin via iontophoresis and the application of an external electrical field [66,67].

Although the safety and efficacy of PCA for children has been well established and PCA for children is quite widely available [68,69], critically ill children as well as very young and/or intellectually disabled children may lack the appropriate cognitive function necessary for self-administration of analgesic medication. PCA by proxy via parent/nursing-controlled analgesia (PNCA) can be effectively implemented through proper education of the proxy and careful monitoring for pain, side effects, and adverse events [70,71,72,73].

#### 2.2.3. Non-Opioids

##### Acetaminophen

Acetaminophen is a synthetic, non-opiate, centrally acting analgesic and antipyretic derived from p-aminophenol [74,75]. Its high therapeutic index and efficacy to safety ratio makes it an attractive and widely used option for PICU mono and multimodal treatment of mild to severe pain as well as fever [74]. Acetaminophen does not affect platelet function or kidney function and is void of gastrointestinal, respiratory, or cardiovascular effects. The IV formulation of acetaminophen was approved for use in the United States in 2010 and has been in extensive use in over 80 countries since 2002 [75]. IV acetaminophen is especially advantageous in the PICU setting for those who have Nil Per Os (NPO) status or are intolerant of enteral feeds. IV acetaminophen achieves analgesic effects within 5 min of administration and reaches higher max concentration (C_max_) at an earlier time (T_max_) than the equivalent dosage administered by oral or rectal route [74,76,77,78,79]. Acetaminophen is metabolized by the liver via glucuronidation, oxidation, and sulfation. Regardless of route of administration, the terminal elimination half-life of acetaminophen is approximately 2 to 4 h in children, adolescents, and adults [74].

A limitation to the use of IV acetaminophen is often its high cost. In terms of cost per dose and total doses dispensed, IV acetaminophen ranks at the top of the list of most expensive medications used in the PICU, along with dexmedetomidine, eculizumab, and botulism immunoglobulin [80]. The use of IV acetaminophen in the ICU setting has been optimized through multidisciplinary quality improvement projects aimed at reducing the associated medication-related variable costs [80,81].

##### Non-Steroidal Anti-Inflammatory Drugs (NSAIDs)

NSAIDs work by blocking the production of prostaglandins which leads to the reduction of pain and inflammation. There are two types of NSAIDS: Non-Selective NSAIDs (ibuprofen, naproxen, ketorolac, and acetylsalicylic acid) that block two enzymes involved with inflammation--cyclooxygenase-1 (COX-1) and cyclooxygenase-2 (COX-2), and COX-2 (celecoxib) Selective NSAIDs. Most commonly, only the non-selective NSAIDs are used in the PICU. The Cox enzymes catalyze the conversion of arachidonic acid into prostaglandin [82]. The COX-1 enzyme regulates key cellular processes of platelet aggregation, afferent arteriole vasodilation in the kidney, and acid protection of the gastric mucosa. The COX-2 enzyme is an inducible enzyme that increases during inflammatory processes and is present in the brain, kidney, bone, and female reproductive tract [83,84]. NSAIDs are widely used for their analgesic, anti-inflammatory, and antipyretic properties. Ibuprofen has become the NSAID of choice for enteral administration, favored for its efficacy and tolerability profile without the risk of Reye’s Syndrome [85]. Ketorolac (IV and Oral), although not approved by the IV route for pediatric patients, is a frequent choice for post-operative analgesia in the PICU [71,86,87,88].

NSAIDs are favored for their lack of opiate-related side effects of nausea, vomiting, pruritus, respiratory depression, tolerance, withdrawal, sedation, and urinary retention [89]. Metabolized in the liver to inactive metabolites via oxidation and conjugation, NSAIDs are excreted in the urine [90]. The risks associated with NSAIDS are related to their inhibition of prostaglandin synthesis. Careful patient selection requires avoidance of its administration to those at risk of acute renal failure, post-surgical bleeding, or gastrointestinal toxicity. In the pediatric population, NSAIDS have not been shown to increase the risk of renal and bleeding complications for both cardiac and non-cardiac post-operative care [91].

##### Gabapentin

Though gabapentin is usually considered an antiepileptic, it is useful for opioid-sparing pain management. Gabapentin inhibits presynaptic voltage-gated calcium channels (upregulated during surgical trauma) in the dorsal root ganglia and spinal cord, preventing release of excitatory neurotransmitters [92]. There is no well-established consensus on the appropriate dosing regimen for gabapentin in the pediatric population. In adults, administration every 2 h in the post-operative period has demonstrated effectiveness, especially for those at highest risk of severe pain [92,93].

##### Ketamine

Ketamine is an *N*-methyl-D-aspartate (NMDA) antagonist which has been available since the mid-20th century. NMDA receptors have been shown to play an important role in central sensitization formation, so it makes sense that ketamine is an effective opioid adjunct [92]. Studies have demonstrated that low-dose ketamine use with standard opioid administration decreased opioid use and led to improved quality of pain, with no reported serious side effects especially in patients for whom higher postoperative pain scores were anticipated and patients with chronic pain and/or opioid dependency [92,94]. A retrospective study of 32 mechanically ventilated children reported that drug rotation with ketamine in mechanically ventilated children with opioid tolerance is feasible and seems to reduce the rate of fentanyl infusion [95].

##### Alpha 2 Agonists

The analgesic effect of alpha 2 agonists like dexmedetomidine and clonidine results from stimulation of α2-adrenoreceptors that are located both in the spinal cord and supraspinal region [92]. Adult studies have shown that both alpha 2 agonists reduce opioid consumption in the perioperative period with dexmedetomidine being more effective [96]. Clonidine is a potent analgesic adjuvant that improves the analgesic effects of anti-inflammatory agents and has significant antinociceptive effects when it is combined with opioids, ketamine, and local anesthetics [97]. Clonidine has been shown to be a highly effective analgesic agent in the perioperative period for adenotonsillectomy and ophthalmological surgery in children [98,99,100].

Dexmedetomidine has not been shown to reduce postoperative opioid requirements or pain scores in children. In combination with opioids, it has been shown to increase time to first analgesic, reduce the need for additional rescue analgesia doses and significantly lower rescue medication requirements for nausea and vomiting postoperatively [101,102].

#### 2.2.4. Multimodal Pain Management

In the practice of pediatric pain management, a multimodal approach considering analgesics (opiate and non-opiate), local and regional analgesia is preferable to monotherapy. Ketamine, gabapentin, and alpha2 agonists have been shown to produce profound antinociceptive effects and reduce opioid use when combined with acetaminophen, NSAIDs, and opioids in both adult and pediatric literature [21,92,103,104]. Non-opioid analgesics have a ceiling effect when used alone. A combination of acetaminophen and NSAIDS have shown favorable results in decreasing opiate requirement and pain scores, avoiding the ceiling effect which can occur when each is utilized alone [103,104,105,106]. Multimodal analgesia has been demonstrated to be particularly effective in managing the pain associated with acute vasoocclusive crises in children and adults with sickle cell disease [107]. Continuous naloxone infusion is used in the PICU for treatment of opioid-induced pruritus.

### 2.3. Neuraxials and Peripheral Nerve Catheters

Regional anesthesia, when used in combination with general anesthesia, has been shown to reduce the amount of opioids and inhaled anesthetics used intraoperatively [103,108]. The European prospective observational multicenter cohort study (APRICOT) prospectively collected data for over 31,000 pediatric procedures performed in 33 countries. Regional anesthesia was performed perioperatively for 4377 surgeries, with central and truncal blocks representing 43 and 42%, respectively. Caudal blocks were the most common (77%) central block [108]. Ultrasound guidance with proper training can decrease time to perform block, increase success rate, hasten onset time, prolong block duration, and decrease volume of local anesthetic required [109,110]. Continuous epidural anesthesia (CEA) (caudal, lumbar, and thoracic) is a common route of post-operative analgesia in the PICU. In the premature and term infants, CEA has been shown to decrease opiate requirements, duration and need for ventilatory support as well as duration of PICU stay [111,112,113,114]. Patient controlled epidural anesthesia (PCEA), popular in adults, is another option that has been used with success in older children and adolescents [103,115].

## 3. Sedation

Sedation in the PICU setting is especially challenging and poses issues that are unique. Mechanical ventilation presents the most significant challenge, balancing sedation to synchronize the patient’s native work of breathing with that of the ventilator. The optimal condition for the non-neuromuscularly blocked patient would be that she is easily arousable or conscious, comfortable, and breathing in sync with the ventilator, a state that can be referred to as the Goldilocks Zone (not too deep and not too light) [21]. Balancing the depth of sedation is important because undersedation can lead to dislodged intravascular access and catheters, unplanned extubation and potentially staff or patient injury. Oversedation, on the other hand, can lead to unstable hemodynamics, respiratory depression and the potential for failure to extubate. Prolonged intubations pose an increased risk for muscle deconditioning, delirium, cognitive impairment, tolerance, withdrawal, and PICU-acquired complication (PACs) [1,6,21,116,117,118,119,120]. Naloxone and flumazenil boluses are used for reversal of unwanted opioid- and benzodiazepine-induced respiratory depression and oversedation.

### 3.1. Sedation Assessment

The State Behavioral Scale (SBS), the COMFORT scale, the COMFORT behavior scale (COMFORT-B scale), and the Richmond Agitation Sedation Scale (RASS) have been validated in PICU patients.

The SBS follows 8 behavioral dimensions in mechanically ventilated children: respiratory drive, response to ventilation, coughing, best response to stimulation, attentiveness to care provider, tolerance to care, consolability, and movement after being consoled [63]. Each behavioral marker can be assigned three–six levels, their sum intended to follow the patient along the sedation-agitation continuum. The SBS, intended for use along the entire pediatric age spectrum, purposely excluded descriptors which would not be met by children <6 years: ability to communicate, follow commands, and attempt to sit or climb out of bed [63]. Similarly, physiologic vital signs (heart rate, blood pressure, respiratory rate) were also excluded, as they have been shown to have low sensitivity and specificity for quantifying degree of sedation/agitation [63,121,122,123]. SBS score ranges from −3 (unresponsive) to +2 (agitated) with a target of 0 intended to achieve the “Goldilocks zone” of awake and able to calm. The SBS should be paired with pain assessment scores every 4 h as well as prior to any interventions that would alter the analgosedative level [63].

The COMFORT scale is an observational scale that measures eight clinical parameters to determine a critically ill child’s level of distress. Unlike the SBS, the COMFORT scale contains two physiologic parameters and six behavioral dimensions: heart rate, mean arterial pressure, alertness, calmness, respiratory response, movement, muscle tone, facial expression. Heart rate and blood pressure, despite being reliable determinants and markers for sedation, have been shown to be of limited reliability and validity as determinants of the total COMFORT score [123]. The COMFORT-B scale eliminates these two physiologic parameters, identifying scores of ≤10 as oversedation and ≥23 as undersedation. A limitation of this scale, however, is that the scores of 10–23 are not predictive of adequate depth of sedation. Thus, the COMFORT-B scale is commonly used in conjunction with other observational scales, some requiring nursing input (Nurse Interpretation of Sedation Score) [21,124].

The RASS is an agitation and sedation scale which has been validated for both adults and children (intubated and non-intubated) in the critical care setting [125,126,127]. The RASS is a 10-point scale ranging from −5 (unresponsive) to +4 (combative) with 0 being alert and calm [125,126,127]. For neonates and children with cognitive or developmental limitations for whom level of arousal is difficult to assess, the original RASS has been adapted to replace eye contact with eye opening when the RASS is −1 to −3 [127].

An important limitation of all of these assessment scales is their inability to be used for children with neuromuscular blockade. For these patients, the Bispectral Index (BIS) monitor has been shown to correlate well with the COMFORT scores, particularly for those with deep sedation [128]. BIS should be considered for patients who are not able to mount a response, taking into account however that BIS values are vulnerable to inaccuracy related to some medications (ketamine, nitrous oxide), hemodynamics, electrical interference from other monitors, and temperature shifts [129].

### 3.2. Sedatives

#### 3.2.1. Benzodiazepines

Benzodiazepines, particularly midazolam, are the most commonly used sedative in PICUs, used not only for sedation but also as muscle relaxants, anticonvulsants, amnestics, and hypnotics [21,130]. Benzodiazepines work on the on the gamma amino butyric acid (GABA)-A receptor, reducing the excitability of neurons thereby producing a calming effect on the brain [131,132]. Benzodiazepines demonstrate a dose-dependent respiratory and myocardial depressant effect and may cause hypotension, particularly in the hypovolemic neonate [30,133,134].

##### Midazolam

Midazolam is a short acting benzodiazepine that is the sedative of choice in most PICUs [30,130]. Midazolam can be administered orally (PO), rectally (PR), intramuscularly (IM), intranasally (IN) and intravascularly (IV). Fastest onset is via the IV route at 1–3 min, followed by IM/IN at 5–10 min and then PO/PR at 10–30 min [5]. IV midazolam has a duration of action of 45 to 60 min. When used for sedation, the IV bolus dose is 0.05 to 0.1 mg/kg (maximum of 20 mg per dose) and the maintenance infusion ranges from 0.05 to 0.12 mg/kg/h. For refractory status epilepticus, a bolus of up to 0.2 mg/kg with a maintenance infusion up to 0.4 mg/kg/h is common [135,136]. Midazolam is metabolized by hydroxylation to an active metabolite (1-OH midazolam) and subsequently undergoes glucuronidation to 1-OH-midazolam-glucuronide, also an active metabolite, which is then renally excreted. Patients with renal insufficiency or failure may experience oversedation with midazolam if there is no dose adjustment [137]. Adverse effects include tolerance, dependence, and withdrawal; there is also a risk of paradoxical hyperactivity when given as a bolus dose [116,117].

##### Lorazepam

Lorazepam is a long-acting benzodiazepine that is commonly used in the PICU for its antiepileptic and anxiolytic properties. Lorazepam can be administered PO, IM, and IV at the same dose of 0.5 mg/kg (maximum of 2 mg per dose) [5,21]. IV lorazepam is dissolved in propylene glycol which at high levels can produce metabolic acidosis and renal dysfunction. Therefore, IV lorazepam is not used as an infusion [21,138,139]. Oral lorazepam plays an important role for converting and weaning patients from long term midazolam infusions to an oral regimen [21,140].

##### Diazepam

Diazepam is a long-acting benzodiazepine that is administered for its anxiolytic, muscle relaxant, and antiepileptic properties. It can be given PO or IV [5]. IV Diazepam is highly water insoluble and is dissolved in propylene glycol. Diazepam can burn on injection and may cause phlebitis with prolonged infusion. IV Diazepam has an onset of action of 4–5 min and a duration of action of 60 to 120 min [141]. Diazepam is metabolized in the liver—it is N-demethylated by CYP3A4 and 2C19 to the active metabolite N-desmethyldiazepam, and is hydroxylated by CYP3A4 to the active metabolite temazepam. N-desmethyldiazepam and temazepam are both further metabolized to oxazepam, and all are eliminated via glucuronidation. These metabolites are active, accumulate in renal failure, and lead to a half-life of diazepam which can range from 20 to 120 h [142,143].

#### 3.2.2. Barbiturates

Barbiturates are useful for their antiepileptic properties and their ability to control intracranial hypertension. Similar to benzodiazepines, barbiturates act primarily via GABA agonism [144,145]. Respiratory depression and hypotension can occur with barbiturates and caution should be taken when weaning from prolonged infusions in order to avoid withdrawal.

##### Sodium Thiopental

Thiopental is short-acting and is used more commonly in the operating room for the induction of general anesthesia and in the pediatric critical care setting for neuro-protective intubations. Its peak onset is 1–2 min and the duration of action is 30 min [146]. The dose is age-dependent with infants requiring much higher induction doses of 5–8 mg/kg compared to children, teens and adults at 3–4 mg/kg [144,147,148]. Thiopental is metabolized by hydroxylation and oxidation via hepatic metabolism and it has a long elimination half-life of up to 12 h [144,149]. It is important to note that thiopental is no longer available in the US as its manufacturer embargoed its import to protest its use for death by lethal injection.

##### Pentobarbital

Pentobarbital is a long-acting barbiturate used for sedation, status epilepticus, and the treatment of refractory intracranial hypertension after severe traumatic brain injury. When administered via the IV route, pentobarbital has an onset of action at 5 min, peaks at 15 min and has a duration of action >6 h. Pentobarbital undergoes hepatic metabolism via cytochrome p450-induced hydroxylation and glucuronidation [144]. Pentobarbital has an elimination half-life of 12–24 h [144]. Pentobarbital therapy can be effective for severe traumatic brain injury (TBI) of adults with refractory intracranial hypertension. A study of 55 patients reported a one-year survival at discharge of 40% with good functional outcomes in 68% of survivors at 1 year [144]. In pediatric patients with TBI, pentobarbital reduced ICP to below their treatment threshold of 20 mm hg in 28% of patients within 6 h. Side effects of myocardial depression with resultant hypotension, necessitating pressors, should be anticipated during treatment [150]. Pentobarbital is a valuable option for refractory intracranial hypertension not amenable to surgical intervention [151]. 

#### 3.2.3. Alpha Agonists

Alpha agonists are often used alone or as adjuncts to sedative, opiates, or benzodiazepines [92]. The sedative-hypnotic effects of alpha 2 agonists are the result of inhibition of norepinephrine release from noradrenergic receptors in the locus ceruleus area of the brainstem [152]. The advantage of alpha 2 agonists is lack of respiratory depressant effect, advantageous in extubating children on prolonged sedatives [153,154,155]. Bradycardia, bradyarrhythmia, and hypotension, not typically requiring pharmacologic intervention, should be anticipated [154,156].

##### Clonidine

Clonidine can be delivered PO, IV, transdermally, or epidurally. Typical clonidine dose is 4 to 5 mcg/kg. Clonidine IV bolus dose is 1 to 2 mcg/kg with 1 mcg/kg/hour as an infusion rate. The dose for neuraxial Clonidine is 1–2 mcg/kg. The transdermal patch delivers 0.1 mg per 24 h and is changed weekly [157,158,159,160,161]. Fifty percent of a dose of clonidine is metabolized by the liver to inactive compounds while the other unchanged drug, along with the metabolites, is excreted in urine and stool. The elimination half-life of enteral clonidine is 12 to 16 h; the CSF elimination half-life is 1.3 h [162]. Clonidine and midazolam have comparable profiles for the sedation of ventilated children [156]. Clonidine has been used to wean children from prolonged dexmedetomidine and opiate infusions [163].

##### Dexmedetomidine

Dexmedetomidine is approved for administration only by the IV route but is effective also by the IM, IN, and sublingual route. It is metabolized by the liver via glucuronidation and oxidation and it has no active metabolites; the half-life of dexmedetomidine is 2 h [21,164]. Dexmedetomidine’s sedative effect closely mimics natural sleep, with EEG activity in children resembling Stage 2 sleep [165]. When comparing dexmedetomidine to midazolam and propofol in adults, dexmedetomidine improves arousability and patient cooperation, decreases duration of mechanical ventilation, and shortens time to extubation. Length of ICU stay and mortality rate were similar amongst the groups [15]. In children, dexmedetomidine is synergistic with propofol, decreasing propofol requirements by up to 50% when used for procedural sedation for gastrointestinal endoscopy [166]. An open-label, pilot, prospective, multicenter, randomized controlled trial with dexmedetomidine was designed to evaluate the sedation of mechanically ventilated children. Dexmedetomidine as the primary sedative was feasible, appeared safe, achieved early, light sedation (State Behavioral Scale −1 to +1), and reduced midazolam requirements. There were more episodes of hypotension and bradycardia with the dexmedetomidine group (including one serious adverse event) but no difference in vasopressor requirements [167]. An observational prospective multicenter (9 PICUs) study (PROSDEX) evaluated the efficacy and effects of dexmedetomidine for prolonged sedation (≥24 h) in critically ill pediatric patients. Dexmedetomidine assured comfort, spared use of other sedatives, and attenuated withdrawal and delirium symptoms. The loading dose and infusion dosages of dexmedetomidine were independent risk factors for hemodynamic adverse events [168].

#### 3.2.4. Propofol

Propofol is a GABA agonist and a diisopropyphenol anesthetic that is advantageous in the critical care setting due to its rapid onset (1–2 min), high potency that consistently produces the desired sedative effect, short duration of action (2–8 min), and antiemetic and euphoric properties that lead to more positive patient experience [15,141,169]. The bolus dose of propofol is 0.5 to 1 mg/kg with an infusion rate of 1 to 3 mg/kg/h. In general, the half-life of propofol after an infusion is 30 to 60 min, longer with prolonged infusions as it is highly lipophilic, redistributed from fat stores [15,141,169,170]. Propofol is metabolized by hepatic glucuronidation and hydroxylation [169]. The adverse effects of propofol are pain on injection, vasodilation or negative inotropy leading to hypotension and/or bradycardia, respiratory depression, apnea, hypertriglyceridemia, and pancreatitis [15,141,169,170]. With prolonged infusion rates of >4 to 5 mg/kg/h and use of long-term propofol infusion in pediatric patients, there is a risk of propofol infusion syndrome (PRIS) characterized by lactic acidemia, rhabdomyolysis, dysrhythmias, cardiac arrest, and a high mortality rate (52% in children and 48% in adults) [15,169,171]. Despite the concern for PRIS, propofol is still widely used in PICUs [172,173,174].

#### 3.2.5. Ketamine

Ketamine has both analgesic and sedative properties. Ketamine is a phencyclidine derivative and an NMDA antagonist and is typically used as an adjunctive sedative agent or as part of a multimodal analgesic regimen as discussed earlier [30,94]. Ketamine is rapid-acting and it preserves laryngeal reflexes allowing for spontaneous respirations during procedural sedation. Ketamine is also a powerful bronchodilator which makes it the agent of choice in patients with severe bronchospasm like status asthmaticus [30]. Although ketamine is a myocardial depressant with vasodilating properties, its indirect sympathomimetic activity (stimulating catecholamine release and inhibition of catecholamine reuptake) preserves cardiac output and leads to increase in blood pressure and heart rate [15,30].

Ketamine has a rapid onset of 30 to 60 s when given IV with effective procedural sedation conditions achieved in 1 min and lasting up to 5 to 10 min. Adverse effects of ketamine include sialorrhea, as well as psychotogenic reactions such as emergence delirium, disorientation, hallucinations, and combativeness at higher serum concentrations [30,141,175,176,177,178]. Ketamine is metabolized by the liver to an active metabolite, norketamine; ketamine and norketamine are then further metabolized by the liver to water soluble compounds that are then renally excreted [179].

#### 3.2.6. Antihistamines and Antipsychotics in the PICU

First generation (sedating) antihistamines such as promethazine, trimeprazine, and diphenhydramine have antidopaminergic and anticholinergic actions. These agents are non-specific and act on histaminic, serotonergic, and cholinergic receptors with marked central nervous system (CNS) effects such as hypnosis, sedation, antiemesis, and paradoxical excitation in children [21,180]. Although not commonly used for PICU sedation, they can be adjuncts for the management of acute agitation and delirium [21].

Antipsychotics (haloperidol, chlorpromazine, risperidone, olanzapine, and quetiapine) are useful treatments for refractory delirium [181]. First-generation antipsychotics, such as haloperidol and chlorpromazine, have high rates of extrapyramidal symptoms (EPS) due to their strong dopamine D_2_ antagonism [181]. Haloperidol is popular in the critical care setting because of its IV formulation. Second-generation antipsychotics such as risperidone, olanzapine, and quetiapine have lower rates of EPS and tardive dyskinesia but with long term use they are associated with an increased risk of weight gain, metabolic side effects, and dyslipidemia. Risperidone and quetiapine are often favored for their favorable side effect profile in the treatment of delirium [181,182,183].

### 3.3. Sedation Protocols

**Figure 1:**
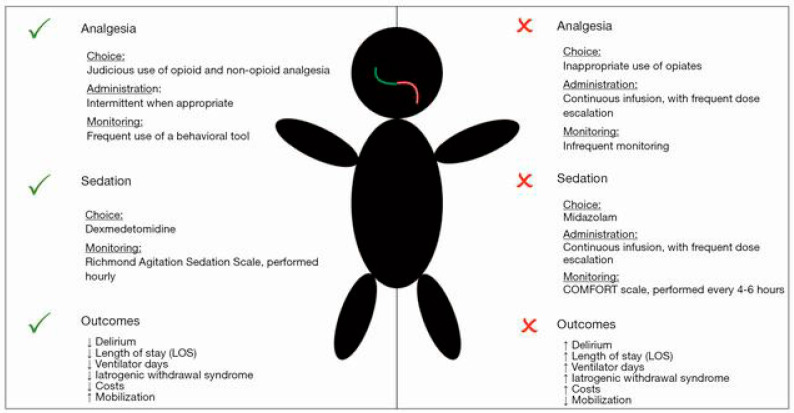
An updated analgosedation approach (left) versus a traditional benzodiazepine-based sedation regimen (right). Republished with permission of NANCY INTERNATIONAL LTD SUBSIDIARY AME PUBLISHING COMPANY, from *Sedation strategies in children with pediatric acute respiratory distress syndrome (PARDS)*, Rosenberg, L. and Traube, C., Annals of Translational Medicine, Vol 7, No 19, 2019; permission conveyed through Copyright Clearance Center, Inc [185].

The efficacy of protocolized sedation is unclear in pediatrics due to the paucity of randomized, controlled clinical trials [184,185]. Sedation protocols for mechanically ventilated PICU patients have been shown to improve PICU resource utilization, decrease the benzodiazepine and opiate days and increase the amount of ventilated-time awake [4,186]. Nurse-implemented, goal-directed sedation protocols, however, have not been shown to decrease the days of mechanical ventilation. Figure 1 compares an updated and more appropriate analgosedation approach to a traditional benzodiazepine-based sedation regimen.

### 3.4. Daily Sedation Interruption (DSI)

Daily sedation interruption (DSI) has been used to mitigate the negative effects of oversedation and prolonged benzodiazepine use. A study comparing daily interruption versus continuous sedative infusions in mechanically ventilated children demonstrated that the length of mechanical ventilation, duration of intensive care unit stay, total dose of midazolam, and average calculated cost of the therapy were significantly reduced in the interrupted [187]. Conversely, a multicenter, randomized controlled trial comparing daily sedation interruption (DSI) plus protocolized sedation (DSI + PS) to protocolized sedation only (PS) found that interruption did not improve clinical outcome in critically ill children and was associated with increased mortality. The value of interrupted daily sedation still remains unclear. The differences in outcomes between these two groups could be attributed to differences in patient populations, medical conditions, and baseline ICU clinical practices [21,188]. These authors recommend, based on clinical experience, that should DSI be implemented, it be done with caution.

### 3.5. Drug Cycling

The rationale behind drug cycling is that cycling sedative agents will limit the patient tolerance and tachyphylaxis to the sedatives, decrease the body total deposit of sedatives, and subsequently decrease emergence time when the patient is ready for extubation. Some PICUs use drug cycling or drug rotating to decrease the adverse effects of continuous sedation, despite minimal data to support this practice [21,189,190].

### 3.6. Delirium in the PICU

The American Psychiatric Association’s fifth edition of the Diagnostic and Statistical Manual of Mental Disorders (DSM-5) define delirium as a disturbance in attention (i.e., reduced ability to direct, focus, sustain, and shift attention), awareness (reduced orientation to the environment), and cognition (e.g., memory deficit, disorientation, language, visuospatial ability) that develops over a short period of time (a few hours to a few days), tends to fluctuate in severity in the course of a day, is not better explained by a pre-existing, established, or evolving neurocognitive disorder, and there is evidence that the disturbance is a physiological consequence of another medical condition, substance intoxication, withdrawal syndrome, or multifactorial [191]. Delirium in the PICU leads to prolonged mechanical ventilation, increased length of PICU admission and hospital stay in general, higher rates of morbidity and mortality, and increased medical care cost [184,185,186,187,188,189,190,191,192,193,194,195,196,197,198]. Prolonged exposure to sedative medications, higher depths of sedation, young age, baseline developmental disorders, and PICU environmental factors are risk factors for delirium [185,192,193,194,199]. Diagnosing delirium can be especially challenging in the PICU setting as the symptoms of cognitive disturbance, hallucinations, and hypoactive delirium are not always able to be verbalized and expressed, especially in the young, preverbal, developmentally challenged patient [21,200,201].

#### Delirium Monitoring and Treatment

Risk factors associated with delirium may be reduced by decreasing medication exposure by measuring sedation depth, avoiding benzodiazepines and anticholinergics as much as possible, and protecting sleep by clustering patient cares, minimizing overhead pages, dimming lights at night, and using ear plugs and eye masks [130,202]. It is important to screen PICU patients regularly for delirium via validated assessment tools.

There are a variety of assessment tools. The Cornell Assessment of Pediatric Delirium (CAPD), considered the standard of care in most PICUs in North America, was validated in children of all ages for mechanically ventilated children [9,185,203]. The CAPD is 94% sensitive and 79% specific [203]. A prospective observation double-blind cohort study to improve the specificity of the CAPD, to allow for accurate detection of delirium in developmentally delayed children admitted to the PICU, demonstrated that, when used in conjunction with RASS score fluctuation, the CAPD is a sensitive and specific tool for the detection of delirium in children with developmental delay and this allows for reliable delirium screening in this hard-to-assess population [204].

The Pediatric Confusion Assessment Method for the ICU (pCAM-ICU) for age five years and older and the Preschool Confusion Assessment Method (psCAM-ICU) are also validated in children and have high reliability, with a high sensitivity and specificity of 83% and 99%, respectively [200]. The Sophia Observation withdrawal Symptoms scale-Pediatric Delirium scale (SOS-PD) is another validated tool for delirium screening in critically ill children, with measurement properties comparable to the CAPD, psCAM-ICU, and the pCAM-ICU and an overall sensitivity of 92.3% and specificity of 96.5% [205,206,207].

Treating delirium may involve non-pharmacologic and pharmacologic approaches. A retrospective matched cohort study in the PICU suggests that antipsychotics may be more effective for early vs late-onset delirium refractory to non-pharmacologic treatment [21,181]. Table 1 depicts a comparison of pediatric delirium screening tools [30].

### 3.7. Withdrawal

Prolonged infusions of analgosedative agents, especially opioids and benzodiazepines, leave patients at high risk of developing withdrawal with discontinuation.

A multidimensional predictive model identified younger age, preexisting cognitive or functional impairment, higher nursing workload, >one-to-one nurse staffing, ≥3 preweaning sedative classes, higher preweaning mean daily opioid dose, higher sedative doses and longer exposure periods are risk factors for iatrogenic withdrawal syndrome in critically ill children [208].

Although opioid withdrawal syndrome can occur as early as two–three days, it tends to occur after five days of continuous opioid administration [209,210,211]. The most common symptoms of opioid withdrawal include diarrhea, vomit, sweat, and fever [209]. A mean benzodiazepine dose of 0.35 mg/kg/h increases the risk of developing withdrawal symptoms which include anxiety, insomnia, irritability, restlessness, hand tremors, muscle spasms, and seizures [210,211]. The incidence of withdrawal after >3 days of infusions is cited as 37%, with high benzodiazepine dosing as a predictor. Other studies report withdrawal in up to 50% after 48 h, increasing to up to 80% after >5 days continuous infusion [211,212].

#### Withdrawal Monitoring and Treatment

Differentiating between delirium and withdrawal can be difficult using the current assessment tools which rely on clinical, physiologic, and behavioral signs [213]. Patients at high risk for withdrawal should be identified prior to initiating the infusion [209,210,211]. There are three validated iatrogenic withdrawal assessment scales specific for the pediatric population: Withdrawal Assessment Tool-1 (WAT-1) scale, the Opioid and Benzodiazepine Withdrawal Score (OBWS), and the Sophia Observation Withdrawal Symptoms (SOS) [212]. A major limitation of all these scales is their failure to accommodate for age and development. For example, the OBWS includes the Moro reflex which is a significant manifestation of withdrawal in newborns but disappears by three months of age. Future research needs to be directed to create new assessment tools or adapt these existing scales to developmental stages [212,214].

Dexmedetomidine and clonidine have been shown to minimize the manifestations of iatrogenic withdrawal symptoms [212,215]. Enteral and parenteral methadone reduce the risk of iatrogenic withdrawal symptoms produced by opioids [57,212]. Phenobarbital has also been shown to be efficient in alleviating and reducing the intensity of refractory withdrawal from opioids and benzodiazepine [212]

## 4. Neurodevelopmental Outcomes

The majority of the studies assessing neurodevelopmental outcomes of pediatric patients exposed to analgosedative agents have focused mainly on general anesthesia. Brief (<1 h) exposure to general anesthesia has not been shown to alter neurodevelopmental outcome at age five years as compared to awake/regional anesthesia and general anesthesia exposure before age three years and is not associated with deficits in general intelligence [216,217]. Systematic review of studies evaluating neurodevelopmental outcomes and prospectively enrolling children exposed to a single GA procedure compared with unexposed children demonstrated that a single GA exposure is associated with statistically significant increases in parent reports of behavioral problems with, again, no difference in general intelligence [218]. Given key differences between general anesthesia and ICU sedation, such as the duration of exposure and the use of multimodal analgosedative regimen in the ICU, it is difficult to draw conclusions about PICU sedation from these general anesthesia studies.

Cumulative opioid dosing has shown an association with worse cognitive scores in extremely low birth weight NICU infants, even after adjusting for social and neonatal risk factors [219]. It is important to consider that the long-term neurocognitive outcomes of midazolam infusion for neonatal sedation in the intensive care unit is unclear and requires further study [220]. Another study failed to show an association between perioperative and post-operative anesthesia/sedation administration and neurodevelopmental outcomes up to two years following cardiac surgery [221]. A four year follow up of the same cohort demonstrated a small statistically significant association between days on chloral hydrate and Performance Intelligence Quotient (PIQ), and benzodiazepine cumulative dose and lower Beery-Buktenica Developmental Test of Visual Motor Integration (VMI-V). Despite these finding, there was no association between sedation/analgesia drugs and neurodevelopmental outcomes [222].

Adult ICUs have initiated care bundles to address neurocognitive patient outcomes. For example, each arm of the ABCDEF care bundle (Assess, prevent and manage pain; Both spontaneous awakening and breathing trials; Choice of sedative and analgesic; Delirium assessment, prevention, and management; Early mobilization and exercise; Family engagement and empowerment) helps prevent and decrease incidence of delirium and has been shown to enhance the rehabilitation potential of adult ICU patients [223]. Pediatric ICUs must continue to follow the adult model by actively working to adapt similar bundles to deliver well rounded patient care in order to optimize analgosedative regimens, engaging patients to participate in higher level physical and cognitive exercises [224].

## 5. Early Mobilization

Study data in critical care medicine suggest that survivors of both adult and pediatric critical illness tend to suffer significant physical, cognitive, and psychosocial morbidities that often lead to delayed recovery, poor rehabilitation, functional impairments, and decreased quality of life [225,226]. Early mobilization, a key aspect of the ICU Liberation ABCDEF bundle mentioned earlier, is gaining in popularity in critical care medicine, with PICUs taking the lead from adult ICUs. In the past, critically ill children were sedated and immobile because of concerns for their physiological fragility and risk of dislodging vital equipment; however, the ICU Liberation movement has led to a major cultural shift [224]. Large scale studies are still needed to accurately determine the outcomes of early mobilization in critically ill pediatric patients as the published data currently available reveal substantially variable outcome measures. Andelic et al., in their study to evaluate whether a continuous chain of rehabilitation that begins with the acute phase could improve the functional outcome of severe traumatic brain injury (TBI) patients, compared to a broken chain of rehabilitation that starts in the sub-acute phase of TBI, found that better functional outcome occurs in patients who receive an early onset and continuous chain of rehabilitation [227]. Jacobs et al. in their study to report the safety and efficacy of a postoperative approach that avoids pharmacologic and physical restraints, and allows liberal physical activity after single-stage laryngotracheal reconstruction in children, demonstrated that, for developmentally appropriate children, postoperative management after single-stage laryngotracheal reconstruction does not require the use of physical and pharmacologic restraints and that older children who are not sedated or restrained and who are allowed liberal physical activity have shorter pediatric intensive care unit and hospital lengths of stay, and a decreased incidence of postoperative adverse events [228].

Even though the data do suggest that early mobilization is safe, feasible, and can be employed in a variety of pediatric critically ill populations, implementing guidelines in the PICU can be quite challenging due to the vast differences in cognitive ability and physical capacity of pediatric patients, the scarcity of pediatric physiotherapy resources, and the great variability of medical diagnoses found in the PICU.

An observational quality improvement project called “PICU up!” studied 200 critically ill children to assess the feasibility of early mobility in the PICU, and demonstrated that implementation of a structured and stratified early mobilization program in the PICU was feasible and resulted in no adverse events. PICU Up! increased physical therapy and occupational therapy involvement in the children’s care and increased early mobilization activities, including ambulation [229]. Simone et al., in their study, examined the impact of an ICU bundle on delirium screening and prevalence as well as described characteristics of delirium cases and found that implementation of an ICU bundle, along with staff education and case conferences, is effective for improving delirium screening, detection, and treatment and is associated with decreased delirium prevalence [207]. Family engagement is also crucial as some data suggest that family involvement helps propel early mobility efforts forward [227]. Bundled interventions that emphasize optimizing sedation such that frequent sedation assessment with validated tools helps guide the titration of analgosedative agents to goal-specific effects, minimizing benzodiazepines, screening for delirium frequently, and promoting sleep in the PICU will transform PICU culture of immobility and reduce the incidence of PICU-acquired complications (PACs).

## 6. Neuromuscular Blockade

Neuromuscular blockade may be a necessary adjunct to analgosedative agents in patients who have serious to severe critical illness as it has been shown to help facilitate endotracheal intubation, prevent ventilator dyssynchrony, reduce barotrauma, facilitate mechanical ventilation in patients with high peak inspiratory pressures not responsive to conventional ventilation, decrease oxygen consumption in severe respiratory failure, minimize ventilator-induced lung injury (VILI), control intracranial pressure spikes in critical traumatic brain injury, control intraabdominal pressure spikes in patients with open abdomens post-operatively, and treat therapeutic hypothermia-induced shivering in post-cardiac arrest critical care [15,230,231,232,233,234,235]. Neuromuscular blocking agents (NMBAs) are selected based on indication, patient’s comorbidities (hepatic or renal failure), and interactions with other drugs that may enhance or prolong their action [230]. Given the major concerns surrounding prolonged deep sedation and immobilization of patients in the PICU, neuromuscular blockade use in critical illness has decreased. There has been a concern for the development of critical illness myopathy (CIM), critical illness polyneuropathy (CIP), or the combination—critical illness polyneuromyopathy (CIPNM) with neuromuscular blockade and glucocorticoid administration in the ICU; however, most studies have shown no consistent relationship between critical illness neuromuscular abnormalities and the use of glucocorticoids and NMBAs [236]. Cisatracurium is more and more the agent of choice for maintenance of muscle relaxation in the PICU because it is a non-steroidal benzylisoquinoline that is broken down via Hoffman degradation independent of hepatic or renal metabolism or excretion and it has been shown to be associated with more rapid spontaneous recovery of neuromuscular function compared with vecuronium; however, there is no evidence that this observed difference in neuromuscular recovery affects outcomes [237,238]. It is also important to note that Cisatracurium is more expensive than all the other neuromuscular blocking agents. Regardless of the choice of muscle relaxant, the recommendation is to monitor the degree of neuromuscular blockade with regular clinical assessment (such as patient’s spontaneous movements or triggering ventilator) and peripheral nerve stimulation using train of four or tetanic stimulation [239,240]. Also, neuromuscular blockade should be discontinued as quickly as safely possible in order to potentially decrease the incidence of prolonged recovery secondary to drug and metabolite accumulation and to potentially decrease the incidence of neuromuscular weakness related to critical illness [240].

## 7. Conclusions

A benchmark of successful sedation in PICU practice should target a situation in which a child is easily arousable or conscious, breathes in synchrony with a ventilator, tolerates procedures and general care routines, and generally appears comfortable. Optimal analgesia and sedation are dependent on the implementation of validated tools to guide the titration of analgosedative agents, and screen for withdrawal and delirium. Optimal sedation should minimize physical and chemical restraints, encourage safe liberal activity, promote restorative sleep, and reduce the incidence of PICU-acquired complications (PACs).

## Figures and Tables

**Table 1:** Comparison of Pediatric Delirium Screening Tools. Reprinted with permission from Beckman E. Analgesia and Sedation. In Buck ML, Manasco KB, eds. Pediatric Self-Assessment Program, 2017 Book 3. *Sedation and Analgesia*. Lenexa, KS: American College of Clinical Pharmacy, 2017 [30].

	DRS-88	DRS-R-98	PAED	pCAM-ICU	psCAM-ICU	CAPD
**PICU population** **(n)**	Med/surg (154)	Med/surg (154)	Med/surg ^a^ (154)	Med/surg, cardiac (68)	Med/surg, cardiac (300)	General (111)
**Age (year)**	1–17	1–17	1–17	≥5	0.5–5	Birth–21 year
**Include mechanical ventilation?**	No	No	No	Yes	Yes	Yes
**Include developmental delay?**	No	No	No	No	No	Yes
**Type of delirium**	Hyperactive	Hyperactive	Hyperactive	Hyperactive Hypoactive	Hyperactive Hypoactive	Hyperactive Hypoactive
**No. of questions or domains**	10	16	5	4	4	8
**Administering provider**	Psychiatrist	Psychiatrist	Anesthesia	Bedside ^b^	Bedside ^b^	Bedside ^b^

^a^ Included postoperative patients, not PICU patients. ^b^ Bedside provider includes nurse, advanced practice provider and physician. CAPD = Cornell Assessment of Pediatric Delirium; DRS-88 = Delirium Rating scale, 1988; DRS-R-98 = Delirium Rating Scale, Revised, 1998; IRR = interrater reliability; PAED = Pediatric Anesthesia Emergence Delirium; pCAM-ICU = pediatric confusion assessment method for the ICU; psCAM-ICU—preschool confusion assessment method for the ICU.
